# Nova Scotia Strong: why communities joined to embrace COVID-19 public health measures

**DOI:** 10.17269/s41997-022-00667-z

**Published:** 2022-07-26

**Authors:** Audrey Steenbeek, Allyson Gallant, Noni E. MacDonald, Janet Curran, Janice E. Graham

**Affiliations:** 1 School of Nursing, Dalhousie University, 5869 University Avenue, Halifax, NS B3H 4R2 Canada; 2 Faculty of Health, Dalhousie University, Halifax, NS Canada; 3 Department of Pediatrics, Dalhousie University, Halifax, NS Canada; 4 IWK Health Centre, Halifax, NS Canada; 5 Department of Sociology and Social Anthropology, Dalhousie University, Halifax, NS Canada

**Keywords:** COVID-19, Qualitative research, Public health, COVID-19 vaccines, COVID-19, recherche qualitative, santé publique, vaccins anti-COVID-19

## Abstract

**Objective:**

To explore Nova Scotian experiences, barriers, and facilitators associated with pandemic public health measures (PHM), including COVID-19 vaccination.

**Methods:**

We conducted semi-structured, individual interviews with Nova Scotians between May and August 2021, during the third wave of COVID-19 cases and provincial lockdown. Participants were recruited across the province from three sectors: decision makers, community leaders, and community members using purposive and snowball sampling. Direct content analysis and thematic analysis were used to identify key themes via the Theoretical Domains Framework.

**Results:**

The experiences of 30 Nova Scotian interviewees clustered around four themes: Communication of PHM, Responsibly Observing PHM: A Community Coming Together, Navigating PHM, and Vaccine Confidence & Hesitancy. Consistent communication of PHM through briefings with the chief medical officer of health and provincial channels reduced misinformation and encouraged PHM compliance. While adherence was high throughout the province, inconsistent enforcement of these measures proved challenging to individuals navigating PHMs. A high level of COVID-19 vaccine confidence and acceptance was identified, and a strong sense of provincial pride prevailed in keeping COVID-19 numbers and transmission low.

**Conclusion:**

This study provides insights into Nova Scotians’ unique experiences with COVID-19 PHM. Provincial public health experts and government leaders communicated PHM with various levels of success, *Nova Scotia Strong*, a sentiment of unity and communitarianism that sprang from public response to tragic events. Future work should aim to include under-represented communities to facilitate broader inclusion.

**Supplementary Information:**

The online version contains supplementary material available at 10.17269/s41997-022-00667-z.

## Résumé

**Objectif:**

Explorer les expériences des Néo-Écossais et les éléments qui entravent ou qui favorisent les mesures sanitaires pandémiques (MSP), dont la vaccination contre la COVID-19, en Nouvelle-Écosse.

**Méthode:**

Nous avons mené des entretiens individuels semi-directifs avec des Néo-Écossais entre mai et août 2021, au cours de la troisième vague de cas de COVID-19 et de confinement dans la province. Des participants de trois groupes (décideurs, responsables locaux et résidents) ont été recrutés dans toute la province à l’aide de sondages intentionnels et cumulatifs. Nous avons utilisé l’analyse directe de contenu et l’analyse thématique pour cerner les principaux thèmes à l’aide du cadre des domaines théoriques (cadre TDF).

**Résultats:**

Les expériences des 30 Néo-Écossais interrogés s’articulaient autour de quatre thèmes : Communication des MSP, Observance responsable des MSP : une communauté qui se rassemble, S’y retrouver dans le dédale des MSP et Confiance envers les vaccins et hésitation vaccinale. La communication cohérente des MSP lors des breffages du médecin-hygiéniste en chef et par les canaux provinciaux a réduit la mésinformation et encouragé l’observance des MSP. Cette observance était élevée à l’échelle de la province, mais l’application incohérente des mesures a rendu difficile pour les gens de s’y retrouver dans le dédale des MSP. Un niveau élevé de confiance et d’acceptation des vaccins contre la COVID-19 a été observé, et un profond sentiment de fierté provinciale a pris le dessus pour réduire le nombre de cas et la transmission de la COVID-19.

**Conclusion:**

Cette étude vient éclairer les expériences uniques des Néo-Écossais face aux MSP liées à la COVID-19. Les spécialistes provinciaux de la santé publique et les chefs du gouvernement ont communiqué les MSP de façon coordonnée et ont bénéficié du sentiment d’unité et de l’esprit communautaire (*Nova Scotia Strong*) qui ont émergé dans la population à la suite d’autres tragédies. Les démarches futures devraient chercher à inclure les communautés sous-représentées.

## Introduction

In Canada, Nova Scotia (NS) is known for its stunning ocean views, quaint seaside towns, and friendly people. Recently, surpassing one million residents, NS offers both rural Maritime charm and a booming metropolitan urban centre (Communications Nova Scotia, 2021a; Statistics Canada, 2022). Underneath this charming veneer, however, is a dark history of settler power and control that amounted to years of systemic racism, prejudice, and inequities disproportionally targeting the Indigenous Mi’kmaq people of NS, the Black NS community, and other equity-deserving groups (Hanrahan, 2008; Sehatzadeh, 2008; Waldron, 2020). According to 2016 census data, Indigenous individuals comprised approximately 8% (~74,040) of the total NS population while individuals of African descent made up 2% (~17,635) (Statistics Canada, 2017).

In NS, wait times for healthcare services are among the longest in Canada (Barua et al., 2019). Gender-based violence is also increasing, and in NS, rates are three times higher among females than among males (Nova Scotia Advisory Council on the Status of Women, 2019). The COVID-19 pandemic and associated public health measures (PHM), specifically the “stay-at-home” provincial mandate, exacerbated violence against women and children (Boserup et al., 2020; Leslie & Wilson, 2020). During the first provincial lockdown, a domestic violence incident that started in Portapique, a rural NS community of about 100 permanent residents, escalated into a mass shooting that left 22 people dead, including 13 women (Mass Casualty Commission, 2022; Public Safety Canada, 2020). This event underscored gender-based violence, temporarily displacing COVID-19 from the news and sparking a “Nova Scotia Strong” response that championed resiliency and communitarianism (Moscovitch, 2020).

During the first waves of the pandemic, NS experienced low COVID-19 cases compared to the rest of Canada, due in part to strict PHM guidance including mask mandates and widely accessible rapid tests (Communications Nova Scotia, 2020). Adherence to PHM was further heightened by trust in key leaders, in particular the Chief Medical Officer of Health (CMoH), who took a primary lead in official public health announcements using evidence-informed, non-partisan guidance, and was strongly supported by the NS premier with catchy slogans like “Stay the Blazes Home.” Morbidity, mortality, and access to personal protective equipment (PPE) were not experienced equally, however, as a notable outbreak at a long-term care facility resulted in the deaths of 53 residents in April 2020 (Nova Scotia Government and General Employees Union, 2020). Long-term care facilities in the province endured chronic, inadequate funding prior to the pandemic (Nova Scotia Government and General Employees Union, 2020; Yacyshyn, 2020), and were slow to adopt adequate PHM and PPE required to minimize disease transmission for high-risk seniors (Nova Scotia Government and General Employees Union, 2020).

These inequities highlight generations of flawed healthcare policies (Oakes & Fleming, 2019), disparate access to healthcare and associated negative health outcomes, and among those most impacted, a distrust for those in positions of authority (Moreira, 2009). The 2013 Ivany Report “Now or Never: An urgent call to action for Nova Scotians” made clear the province’s need to “transform ourselves into a more unified, progressive, creative, and change-oriented society” (2013:viii) (Ivany et al., 2013).

Against this backdrop, we sought to explore Nova Scotians’ experiences adapting to evolving PHM, and identify barriers and facilitators to following provincially mandated PHM, and perceptions towards COVID-19 vaccination in managing the pandemic, with the goal of identifying factors that enable trust in PHM.

## Methods

This exploratory, qualitative study was part of a larger Canadian Institutes of Health Research (CIHR)–funded project aimed at understanding the sociocultural and behavioural factors affecting community response to COVID-19 countermeasures (e.g., quarantine, isolation, vaccination, masking) across Canada. We interviewed Nova Scotian decision makers (DM; e.g., senior health officials, public health experts), community leaders (CL; e.g., health clinic managers, religious leaders), and community members (CM; e.g., public, frontline workers, students) to gain insight into their experiences. This study was approved by the Dalhousie University’s Health Sciences Ethics Board (reference number: 2020-5135), and all participants provided written, informed consent.

### Participants and recruitment

Residents of NS aged 16 and older, fluent in English, French, or Mandarin, and able to provide informed consent, were recruited from DM, CL, and CM sectors to facilitate a diversity of experiences.

Recruitment occurred through purposive and snowball sampling techniques (Noy, 2008; Palinkas et al., 2015). The research team, comprised of academic clinicians and a medical anthropologist, and who were also long-term residents of NS and well connected with equity-seeking groups throughout the province, created an initial contact list of potential participants from various personal and professional networks (i.e., academics, clinicians, administrators, government workers, and community members in NS). The lead research assistant (RA) emailed all potential participants with study details and contact information; a follow-up email was sent one week later to non-responders. Participants who contacted the RA were asked to identify other potential participants from their own networks. All newly identified contacts were then emailed the same study information and invited to participate. Only the RA had knowledge of participants’ names and contact details. With the goal of capturing diverse experiences and accounts, we aimed to recruit 10–12 participants per category (DM, CL, CM), for a maximum 30–36 participants or until data saturation was achieved.

### Data collection

A semi-structured interview guide for DM/CL participants was developed to gather insights into their professional roles and experiences with the development and support of PHM (i.e., that were guided by the provincial PHM strategy), including challenges, barriers, and facilitators. We could not collect age and gender from the DM and CL sector due to REB concerns about participant anonymity.

The CM interview guide included more general questions regarding experiences during the first waves of COVID-19 navigating applicable PHM in participant’s own unique context (Supplementary Files 1 and 2). All interviews were conducted by phone or through Microsoft Teams, and all participants were offered an honorarium of $75 in the form of a cash e-transfer or Amazon gift card. There were no time restrictions applied to the interviews. Audio recordings of the interviews were transcribed verbatim using Otter, an online artificial intelligence software. Generated transcripts were reviewed by a transcriptionist for accuracy and the RA removed any identifying information.

Interviews were conducted between May and August 2021 while NS was experiencing a third wave lockdown that lasted from April 28 to June 2 (Communications Nova Scotia, 2021b). During this time, PHM included the following: limiting socializing to household members, moving schools to virtual learning, closing all non-essential retail stores, and closing restaurants to in-person dining (Communications Nova Scotia, 2021b). The provincial mask mandate remained in effect and over 1.4 million COVID-19 vaccinations were delivered to 70% of the population by the end of August 2021 (Communications Nova Scotia, 2021c), which was comparable with the national vaccination rate (Public Health Agency of Canada, 2021). There were 4257 COVID cases in the province and 28 COVID deaths during this wave (Communications Nova Scotia, 2021b).

### Data analysis

Transcripts were analyzed using a directed content approach via NVivo 12 (Assarroudi et al., 2018). An experienced qualitative RA reviewed transcripts and deductively coded excerpts to domains from the Theoretical Domains Framework (TDF), which maps individual, social, and environmental factors affecting behaviour (Atkins et al., 2017). Thematic analysis was then applied within each domain to identify prominent themes and subthemes by each category. Key themes were reviewed and confirmed by the two study RAs who conducted the interviews to ensure accuracy. The final themes were reviewed and edited by the wider research team.

## Results

### Sample characteristics

A total of 30 participants were interviewed; five were from the DM sector and self-identified as white, while seven were CLs. All but two CLs self-identified as white. The remaining participants were CMs, predominantly female and younger adult, with equal numbers of participants who self-identified as white and other (e.g., Arabic, Black, Chinese). Interviews ranged in length from 27 to 71 min, with a mean length of 50 min. All but three participants resided in urban areas (see Tables 1 and 2 for demographic characteristics).

**Table 1:** Description of DM and CL participants

Participant ID	Self-identity
DM 1	White
DM 2	White
DM 3	White
DM 4	White
DM 5	White
CL 1	White
CL 2	White
CL 3	Middle Eastern
CL 4	White
CL 5	Middle Eastern
CL 6	White
CL 7	White

*DM*, decision makers; *CL*, community leaders

**Table 2:** Description of CM participants

Participant ID	Gender	Self-identity	Age grouping
CM 1	Male	Arabic Canadian	Young adult
CM 2	Male	White	Young adult
CM 3	Female	Black	Young adult
CM 4	Female	Black	Young adult
CM 5	Female	White	Older adult
CM 6	Female	White	Young adult
CM 7	Male	White	Young adult
CM 8	Female	Chinese	Young adult
CM 9	Male	Sri Lankan	Young adult
CM 10	Female	Nigerian	Middle-aged
CM 11	Male	White	Older adult
CM 12	Female	Nigerian	Middle-aged
CM 13	Female	Middle Eastern	Middle-aged
CM 14	Female	Chinese	Young adult
CM 15	Female	White	Middle-aged
CM 16	Male	White	Older adult
CM 17	Female	White	Older adult
CM 18	Female	White	Older adult

*CM*, community members; young adult (16–30); middle-aged (31–64); older adult (65+)

### Themes

Four key themes were identified across the three interview sectors: (1) *Communication of PHM*, (2) *Responsibly observing PHM: a community coming together*, (3) *Navigating PHM*, and (4) *Vaccine confidence and hesitancy*. Themes and supporting interview excerpts follow.

#### Theme 1: Communication of PHM

DM participants voiced a strong commitment and responsibility for successfully communicating and implementing provincial PHM to diverse stakeholders (e.g., employees, service sector, public, etc.). While several DMs expressed their frustration (e.g., constantly evolving evidence, fear of the unknown, and confusing messaging) around implementation, all DMs felt PHM provided a sense of security for Nova Scotians, whereby leaders were seen to be genuinely focused on controlling the pandemic in the province. Additionally, both DMs and CLs emphasized the importance of providing high-quality, accurate, and consistent information by provincial authorities (e.g., CMoH) to successfully implement PHM.

Communication is such a critically important part of a response...the success of Nova Scotia’s response has been making sure Nova Scotians have good information and that we’re building a trusting relationship, because ultimately, we have to rely on people to do the things that we’re asking them and that are required, because we can’t police it all right? (DM 2)

Ensuring information was accessible and up to date helped counteract misinformation. Although several DMs and CLs were surprised that NS had less misinformation circulating on social media than other provinces, several emphasized the need to consistently stay abreast of communication.

If you’re not spreading it [accurate information] widely, and quickly, somebody else will spread misinformation widely and quickly ahead of you. So you got to be doing it. You got to be doing the same avenue that people are spreading misinformation. (DM 4)

While the DMs and CLs acknowledged that some Nova Scotians did share misinformation or conspiracy theories about COVID-19 (and vaccines), the majority of the population followed PHM.

...I think we were expecting and maybe fearful that there would be a lot more, you know deliberate misinformation. And so it was a pleasant situation that there wasn’t, you know, there’s all the other stuff circulating outside in the world...But in terms of how those showed up on our direct channels, it was really very little. (DM 3)

While most DMs deemed the NS PHM communication strategy as successful, others suggested that engagement with underrepresented communities (e.g., BIPOC, immigrant, 2SLGBTQI+) might have made PHM information more relevant and effective. Several CLs acted proactively, reaching out to organizations and communities, and where warranted, translating PHM and vaccination details with newcomers and among those whose first language was not English. Concerned that delays in translation and dissemination may disadvantage certain individuals, some CLs helped translate PHM information into multiple languages. Additionally, several CMs also stressed the importance of including diverse groups (e.g., different age, ethnic, and racialized communities) in knowledge translation to ensure culturally safe information.

I felt the translation was kind of late for other languages. So we got the English, the English guidelines. Now you’re gonna get the translation a month later or a couple of weeks later, which was kind of a big gap...and also identifying some languages was not kind of identified there and we had to make recommendations to include these languages in the translation strategies by Public Health. (CL 5)

I think, it’s just my thoughts though, that another thing that Public Health can do is to try and include people, use racialized populations, and this kind of people in some of the videos and lectures that they produce... Like you want to say wash your hands, use someone from Jamaica, Asia also. So we can all feel it’s all about all of us. (CM 10)

DM, CL, and CM participants expressed their reliance on information conveyed during the regular CMoH and NS Premier’s news briefings. These briefings shared the latest COVID-19 developments, announced revisions to key PHM, and updated vaccination information for the media and public, and became regular sources of information for Nova Scotians in several ways. For example, younger CMs were more likely to engage with local, trusted Instagram accounts (e.g., Halifax Noise, Nova Scotia government), middle-aged CMs were more likely to watch live streams of the briefings, and older CMs were more apt to catch briefing highlights on the evening news. The province’s CMoH became an icon of reliable public health knowledge that united Nova Scotians across the province.

Everybody talks about Dr. Strang and even though he didn’t always have all the information, the credibility that he had and that frequency of the sessions and the media there and all that. But people were dying for that credible source and because he protected us for so long his credibility really grew .... it was really good that we had that kind of strong source. (CL 5)

Provincial and federal websites and associated social media accounts were regularly accessed by CMs across age groups to keep current on PHM.

So at the moment I go to novascotia.ca/coronavirus. I find out all the information that I need. If I have to go further, there’ll be links attached to directories for you to you know, go further and I find it quite easy to navigate and understand. (CM 12)

#### Theme 2: Responsibly observing PHM: a community coming together

A strong sense of collective responsibility and provincial pride in getting through the pandemic was expressed by all participants. Several participants felt that limited community spread was sustained throughout most of the pandemic due to PHM adherence. Additionally, all participants noted that neighbours, relatives, and colleagues often checked in on each other.

...in my neighbourhood, there were a lot of people who would check with their neighbours and say, hey, you know, I’m going to do a grocery run, can I get you anything? Or, you know, how are you doing through this? Do you need anything picked up? That sort of thing and I think it brought neighbourhoods together in checking on each other. (CM 18)

…you know I was laughing actually last night even, right now when you don’t have to wear it [masks] outdoors, we went to a dairy bar close to our house and there must have been 30 people there and everybody had their mask on outside waiting in line with their distancing just like they had at the height of the pandemic, so it was kind of interesting. People you know still following the rules even though they don’t technically have to. (DM 4)

The devastating Portapique mass murders in the early weeks of the province lockdown (Public Safety Canada, 2020) had an indirect effect on PHM adherence. Several participants noted a sense of uneasiness in the province, further highlighting the importance of caring for one another.

So everybody was kind of doing their part and selling things in support of the hashtag Nova Scotia Strong in respect for the Portapique killings. But yeah, like you can really feel when you’re down and out there’s a strong sense of community when tragedy happens, because we are tiny, and everybody’s trying to support each other. (CM 8)

So I think definitely this, during this time, it actually brings everybody together and actually emphasizes and makes my identity as a Nova Scotian much stronger. (CM 14)

#### Theme 3: Navigating PHM

For some CMs, navigating PHM was frustrating, especially as certain PHM (e.g., closures across parks, schools, gyms and restaurants) were applied inconsistently, and with poor rationale, through different pandemic waves. Some restrictions were difficult to implement, and several participants felt that PHM were selectively enforced by authorities, causing occasional dissident behaviour. For example, partying university students violating household number restrictions were fined while people attending an anti-mask rally were not. These frustrations were also felt by CMs working in retail who could not enforce mask mandates yet were tasked with dealing with customers who refused to wear a mask or comply with physical distancing measures.

But then there was all those people on Citadel Hill, like the anti-mask people or whatever. And there was a bunch of police officers there and I didn’t see a single person get a ticket.... Like it just doesn’t feel, like it’s hard for a lot of people to take this seriously when they’re not seeing any consequences put in place. Like I don’t know about you, but I don’t exactly I don’t have $2,000 to just like put over a ticket myself personally. But at the same time, I’m not seeing anybody else actually have to do that. (CM 6)

Although many of the PHM were inconvenient, several participants felt that a sense of collective responsibility contributed to PHM adherence among Nova Scotians. In another student incident, a considerable outlash was sparked when partygoers boasted about their fines on social media. Younger CMs felt that the experience negatively affected how they were being perceived in the province.

I guess, university students, not following rules and you know, holding house parties, it kind of makes you worry a little bit because, you know, people my age are pretty much in that age bracket. So I guess we don’t want to be like, contained in that perception that people, oh, you know, this age of people, they don’t follow the rules or they don’t yeah, they don’t care for the virus at all or anything. (CM 3)

While younger adults were being targeted for non-compliance within the second wave of the pandemic in Autumn 2020, several of the CM participants mentioned that PHM non-compliance occurred across all age groups. However, these interviewees voiced that only a small minority of individuals ignored PHM.

I think everybody just wants to get it over with and, you know, you gotta stay locked down, do your due diligence to make sure we eradicate the virus, but at the same time, you always have those people that will break the rules no matter what. (CM 3)

#### Theme 4: Vaccine confidence and hesitancy

Nova Scotians largely supported COVID-19 vaccination. A few CMs were initially vaccine hesitant, primarily due to uncertainty about safety and long-term adverse effects. These same participants, however, felt that the benefits of vaccination outweighed any concerns and intended to get vaccinated once meeting age eligibility. Positive attitudes associated with vaccination included communitarian values like contributing towards herd immunity, helping to end the pandemic, and seeing friends and family.

I’m excited that they're coming out and I actually just scheduled my vaccination appointment yesterday because they just released like the 18+. I am really happy to see that like when you go to schedule your vaccine appointment in Halifax, so many locations are fully booked. So that tells us that people are like itching to get their vaccines, which is amazing for our communities. (CM 8)

...I don’t think I’ve heard one person that has been kind of worried about the vaccine, you know, because at the end of the day, we all just want to take the vaccine. So we can start to do activities again, you know, see each other in groups and everything. I’m sure there’s a few people that are a little bit worried about it. But at the end of the day, I think our main goal is to be able to do what we love to do again, and we’ll definitely all get the vaccines, definitely. (CM 5)

Several DMs and CLs reiterated similar sentiments, noting the high level of COVID-19 vaccine uptake in the province.

People are anxious to get the vaccine, people are coming to get the vaccine. Nobody seems to be saying that they don’t want it. And if they are there, they’re such a minority that we don’t, I don’t really hear much... they’re anxious to get on with life. And they see having two vaccines as a way to do that. (CL 4)

In NS, the initial vaccine rollout prioritized frontline healthcare workers and older adults, especially those residing in long-term care; age-based vaccine distribution typically followed. Most participants felt that this was fair, supporting the health of essential, high-risk, and/or vulnerable groups in the community.

About the rollout of the vaccine, I think the way it worked is commendable, you know, looking at a population of seniors...making that decision to give them first, it’s top notch. And then coming down gradually by age, by a 5-year I think it’s by 5-year deduction or increment, it’s good. And they gradually got everybody and then also prioritizing health care workers, people who deal with these people every day. (CM 10)

However, some participants felt that certain individuals may have been overlooked in the initial NS vaccine strategy. Some CLs and CMs suggested that retail employees and spiritual leaders should have been prioritized to receive the vaccine in the initial rollout. This sentiment was echoed by some DMs and CLs who received complaints about healthy older adults prioritized over younger, high-risk adults.

anybody that’s doing any kind of frontline work...these people are there every day and serving hundreds of people every day...I think they should have been prioritized too in, you know, keeping the province open. So people can eat, you know, they’re frontline workers, too. Sobey’s, whatever have you, if you’re working there all the time. You don’t know what’s coming through your store. (CM 15)

…people with cancer, people with children with different diseases, you know there was always things that people thought that should have been done first. (DM 4)

Unlike the provincial PHM communication strategy, several DMs and CLs did express concern about the provincial vaccine communication strategy. Several DMs were developing strategies to support uptake of the vaccine early in the pandemic. Much like communicating PHM, DMs emphasized the importance of ensuring the public had access to quality information to inform vaccination decisions, and the efforts required to develop these resources. The high vaccination uptake rates seen across the province suggest that the implementation strategies were successful.

...last summer in anticipation that vaccines were in development, and we would need to do a major vaccine campaign, they did look, and I think they collaborated with other provinces and maybe the federal government too on research around what, you know, what kind of language people would be responsive to, and what kind of language they, you know, made them not feel good about vaccines and vaccination. So they used some of that research to inform their marketing campaigns... (DM 3)

...some good feedback on that social media and the fact that, that we’re still getting good uptake in vaccine, even in the younger age demographics, to me, again, is indication that what we’re doing is working. (DM 2)

## Discussion

From the perspectives of those developing and supporting provincial PHM and from community members affected by PHM, this study explored Nova Scotians’ experiences with COVID-19 PHM, including vaccination. Our findings suggest a high level of compliance with PHM across the province, a strong sense of provincial pride in keeping COVID-19 cases low, and positive views towards the COVID-19 vaccine as a means of ending the pandemic. Suggested improvements for managing the pandemic and associated PHM included taking diversity into account when developing PHM, translating PHM into additional languages, and including essential retail workers and religious/spiritual advisors in initial vaccine rollout.

We suggest that two critical incidents that occurred (in a 2-week period) during the first lockdown, the deaths of 53 institutionalized older adults and 22 victims of the Portapique shootings, sparked an altruistic communitarian “Nova Scotia Strong” attitude that prevailed during the first 2 years of the pandemic. This campaign served to strengthen an existing feeling of collective responsibility (Statistics Canada, 2017) among Nova Scotians to comply with provincial PHM. In resilience research that focused on the aftermath of the 9/11 terrorist attacks on the World Trade Center in the United States, the authors found multiple examples of resilience and recovery even during encounters with mass trauma (Bonanno et al., 2005), while other research found that peoples’ moral behaviours (e.g., kindness, teamwork, caring) increased after the 9/11 tragedy (Peterson & Seligman, 2003). These collective values, social responsibilities, and communitarianism that were experienced in NS may have played a role in supporting PHM compliance and, subsequently, minimizing COVID-19 transmission throughout the province. Prior to COVID-19, PHM have been criticized for promoting individual priorities over social-level values, as social connectedness is essential for living through public health emergencies (Wulff et al., 2015).

With respect to COVID-19 vaccination, we identified a high level of acceptance and intended uptake among all participants. This finding is consistent with national data from Spring 2022, noting that 85% of the province’s population received two doses of a COVID-19 vaccine and 51% received both doses and a booster (Public Health Agency of Canada, 2022), with uptake well above the national average (Public Health Agency of Canada, 2022). COVID-19 vaccination rates were considerably higher than rates in previous vaccination campaigns in the province. For example, since 2010, seasonal influenza vaccine has typically had a 35–40% uptake, and the 2009–2010 pandemic influenza (‘swine flu’/H1N1) vaccine saw only 58% of the population vaccinated (Nova Scotia Department of Health and Wellness, 2018; Statistics Canada, 2015). The severity and unknown factors of COVID-19 illness, more interpersonal communication and subsequent trust in provincially mandated PHM (Bollyky et al., 2022), along with a collective “Nova Scotia Strong” pride may have also contributed to controlling transmission and to vaccine acceptance in the province.

### Limitations

Data collection occurred during a significant COVID-19 outbreak in the province, requiring online individual interviews rather than focus groups. Additionally, the unique experience of a small province, fewer case numbers, and limited community spread may limit the transferability of these findings to other Canadian settings. While our sample is representative of NS’s dominant demographic, it does not capture the voice of under-represented communities. Our non-white participants were predominantly university or high school students, who provided thoughtful critiques of the PHM and vaccine distribution plans but may not have been representative of disadvantaged populations. We also did not collect data about income level or socioeconomic status. Additionally, we did not collect age and gender data from our DMs and CLs due to REB concern surrounding participant anonymity. Time constraints limited sufficient ethical engagement with Mi’kmaq communities in NS, and subsequently, our institutional REB did not approve our study to include Indigenous participants. Future research should aim to include partnership with Indigenous and other equity-deserving communities (e.g., individuals with disabilities, the 2SLGBTQI+ community, people who experience homelessness, etc.).

## Conclusion

This study provides a view of Nova Scotians’ experiences with the COVID-19 pandemic PHM. Despite limitations, qualitative findings from our participants showed that overall, many Nova Scotians favoured following provincially mandated PHM rather than more individualistic libertarian conduct expressed in some other pockets of the country (Chum et al., 2021; Lang et al., 2021). Importantly, Nova Scotians relied on trusted COVID-19 information sources, considered to be independent of politics and social media. We suggest that a shared sense of responsibility to their community was heightened perhaps by the Portapique murders, an incident that prompted widespread reflection on what matters most—a safe and responsible community. Public health and government leaders appealed to and negotiated with Nova Scotians in a manner that united rather than divided along political lines. A critical and pivotal two weeks, early in the pandemic, may have reinforced strong communitarian values and trust in provincial mandated public health measures.

## Contributions to knowledge

What does this study add to existing knowledge?

• We identified that public health measures communication from credible information sources was vital to Nova Scotians’ compliance with PHM.

• Shared community values may have also sustained low case numbers and transmission, and high uptake and acceptance of COVID-19 vaccines in the province as compared to other areas of Canada.

• Inconsistent enforcement of PHM and focus on English-speaking and Caucasian communities were identified challenges.

What are the key implications for public health interventions, practice or policy?

• Inclusive, consistent, clear communication is crucial for engaging all community members in observing public health measures.

• Future public health practice needs to ensure messaging is easily accessible for all members, especially those in under-represented communities.

## Supplementary information

ESM 1 (PDF 102 kb)

ESM 2 (PDF 119 kb)
