# Supplementary material for: Nova Scotia Strong: why communities joined to embrace COVID-19 public health measures
Source: Can J Public Health. 2022 Jul 26;113(Suppl 1):4–13. doi: 10.17269/s41997-022-00667-z (PMC9321281; doi:10.17269/s41997-022-00667-z)
Supplement: Supplementary file 1 — (PDF 102 kb) [file 41997_2022_667_MOESM1_ESM.pdf]

Key informants involved in developing communication strategies/campaigns about public health measures against COVID-19

Interview guide

Lead 1: Personal and professional introduction

1- As a start, I would like you to tell me a little about yourself (e.g. your education, your role in your institution, how long you have been working in this role, etc.)

2- How has your role and responsibilities changed since the start of the COVID-19 pandemic?

Lead 2: Communication about public health measures to contain the spread of the COVID-19 and sources of information about the pandemic

Since the beginning of the COVID-19 pandemic, there have been several public health measures implemented to help contain the spread of the infection in Nova Scotia. Part of your work as a (*position and institution*) has been to develop strategies and materials to communicate these public health measures to the public.

3- Can you speak about how these public health measures were communicated to the public and how you decided on these communication strategies (e.g., channels, messages, spokesperson)?

4- Can you speak about any feedback you received regarding these public health communications?

5- What are your thoughts about the effectiveness of these communication strategies?

6- Can you give me an example of one campaign or communication intervention that went well and one that went wrong?

7- What are the challenges or the difficulties that you face in your work of information communication about the COVID-19?

8- What are the particular challenges or difficulties in terms of information communication about COVID vaccination?

9- What are the sources of information that you use in developing your communication strategies?

Lead 4: Perceptions of the public on COVID-19 and on COVID vaccines

10- In your view, what do people think and say about the pandemic? What are their main questions or concerns? And about COVID vaccine? How do you seek information about this?

11- Have you noticed differences between individuals and groups (e.g. based on sex, age, socioeconomic background, ethnic origin, etc.) both for the pandemic and vaccines?

12- WHO has noted that there is an infodemic on top of the pandemic. That is an overabundance of information (good, wrong and misleading). What are your thoughts or perceptions about the communication environment in (*setting*)? What type of conflicting information have you had to deal with?

13- In your view, how do the media (traditional and online) influence the perceptions of the public about COVID-19? How are you collaborating with media in your work?
