# Supplementary material for: Nova Scotia Strong: why communities joined to embrace COVID-19 public health measures
Source: Can J Public Health. 2022 Jul 26;113(Suppl 1):4–13. doi: 10.17269/s41997-022-00667-z (PMC9321281; doi:10.17269/s41997-022-00667-z)
Supplement: Supplementary file 2 — (PDF 119 kb) [file 41997_2022_667_MOESM2_ESM.pdf]

Community members

Interview guide

Lead 1: Sociodemographic characteristics

1- As a start, can you to tell me a little about yourself (e.g. your age, your gender, your ethnic origin, your marital/family situation, the community in NS you live in, your occupation, etc.)?

Lead 2: Perceptions and experience of the COVID-19 pandemic

2- I would like to know a little about your experience of COVID-19. How has COVID-19 impacted your life and the life of your family and friends? If so how what were the impacts?

3- Have you or your family been negatively impacted by COVID-19 social media messaging, racism, stereotypes? If so can you give some examples of these negative experiences?

4- In your opinion, have the Portapique killings impacted the COVID-19 perceptions and experiences for people in Nova Scotia? For the people in your community? If yes why so?

5- How do you feel about this disease? What are your main concerns?

Lead 3: Communication about public health measures to contain the spread of the COVID-19 and sources of information about the pandemic

Since the beginning of the pandemic, there have been several public health measures implemented to help contain the spread of COVID-19 in (*Nova Scotia*).

6- What do you think about these measures?

7- What are your thoughts or perceptions about how the public health measures against COVID-19 were communicated to the public by the authorities? How were public messages on COVID-19 received by people around you (friends, family members, co-workers, etc.)?
   - How could these communications be improved in your view?

8- How do you personally seek out information about the pandemic? What are the sources of information that you trust the most?

9- To what extent do you use social media as a source of information about the pandemic? What do you like about the information that you find on these platforms? Do you share or regularly post on this topic?

Lead 4: Vulnerable and targeted populations/To be adapted in each setting

Young adults

Young adults have been the target of a lot of public health messaging about the COVID-19. They have also often been accused of not following public health measures to contain the spread of the infection.

10- What are your thoughts and feelings about that?

11- How did this impact you personally? Your friends?

12- How do you think that the government and public health authorities should communicate with young adults about the pandemic (types of messages, channels, speakers, etc.)?

People of Asian descent and other minority groups (e.g., Indigenous, Black community etc)

People of Asian descent and other minority groups have been blamed, in popular discourses, especially during the first wave, of being at the origin of the pandemic.

13- What are your thoughts and feelings about that?

14- How did this impact you personally? Your family?

15- What do you think should have done (by the government, by public health authorities, by the media) to address this issue (types of messages, channels, speakers, etc.)?

Lead 5: Perceptions and intentions regarding COVID-19 vaccines

Vaccines are now available against COVID-19 and since December, public health authorities have actively vaccinated citizens across the country.

16- How do you feel about these vaccines (probe necessity, effectiveness, safety)?

17- What are your intentions and your family members' intentions regarding COVID-19 vaccination? (probe for timing – would like to be vaccinated sooner or later)

18- Because the vaccine doses are limited, public health authorities have decided to target specific groups to receive the vaccine in priority (e.g. healthcare providers and older people). What do you think of this strategy? In your view, who should be prioritized for vaccination after these first two groups?

19- What information would you need to make an informed decision about COVID-19 vaccines? What sources of information about vaccines do you trust? (Explore overabundance of information and infodemic)
